# The Foreign Journals: Medical Jurisprudence

**Published:** 1839-01

**Authors:** 


### MEDICAL JURISPRUDENCE.

*Case of Suspected Infanticide.* By Dr. GRAFF, of Darmstadt.

[A CHILD having been found dead under suspicious circumstances, a medical inspection was directed to be made to determine the cause of death.]

*Externally*, the following appearances were met with. The skin was soft and shrunken, the cuticle easily peeling off. The countenance was wrinkled and presented an aged appearance. About the neck there were marks of lividity, afterwards found to correspond with deep discoloration of the cervical muscles. On many parts of the head, especially posteriorly, there were patches from which the cuticle had been separated. There were no external wounds, with the exception of a small puncture on the outer side of the left foot. This wound contained a little blood, and its margin was somewhat livid and discoloured; but there was no mark of swelling or inflammation in the surrounding structures. The nails of the fingers and toes were not fully developed. The child was of the male sex, but there was no appearance of testicles. The body weighed three pounds. The umbilical cord, about two feet in length, which had been completely torn from the placenta, lay on the left side of the body, and was partially twisted round the under part of the left foot.

*Internally.* On reflecting back the skin of the cranium some blood was found effused: and in the left parietal bone there was a fine fissure about an inch in length, extending to the sagittal suture. Blood escaped through this fissure from the cavity of the cranium. The brain was throughout highly congested: beneath the fissure in the parietal bone there was about a teaspoonful of thick black blood. The muscles of the neck were of a very dark colour. In the chest the diaphragm was found much protruded upwards, the lungs of a dark colour and situated quite posteriorly. Some blood was found extravasated in this cavity. The lungs with the heart and thymus attached readily sank in a vessel of cold water. The lungs separated from the other organs also sank: when divided, they were very firm, not crepitating, and the divided portions sank when placed in water. The foramen ovale was open. No particular appearances were met with in the abdomen. The stomach was small and contained a viscid bloody fluid. The bladder was empty, and the large intestines contained meconium.

From this examination the following opinion was given:

1. *The child was not mature, being about from four to six weeks under the full period.* The proofs of this were: the length, the weight, the shrunken appearance of the body, especially the limbs; the aged appearance of the face; easy separation of the cuticle; the great elasticity of the bones of the cranium; the cartilaginous state of the ribs, and imperfect development of the nails.

2. *The child had either not breathed or breathed but imperfectly.* This was established by the situation, colour, and consistency of the lungs; their sinking in water entire and divided; absence of crepitation; the small quantity of blood contained in them, and the open foramen ovale.

3. *Nevertheless the child was born alive.* The evidence in favour of this was: unusual lividity of the muscles of the neck indicating compression at that part, and confirming the statements of those who found the child with a cloth tied tightly round its neck; the great ecchymosis about the scalp, with the fissure in the parietal bone and extravasation of blood beneath. Some rare cases are on record, in which under difficult labour the cranial bones of the child have become fractured by the muscular contraction of the uterus alone; but the occurrence of such an accident is altogether improbable if not impossible, in cases where, like the present, the child is immature, the cranial bones elastic, and the sutures yielding. If the fracture and extravasation occurred after birth, it follows that the child must have been living; for otherwise it is impossible to admit that such an extravasation could have taken place. Another proof of live birth exists in the fact of blood having been found effused in the chest. In some instances, twisting of the umbilical cord around the neck may lead to asphyxia; but this view was here inadmissible, because when the body of the child was found, instead of the umbilical cord *a cloth was tied fast around the neck.*

Allowing that the preceding facts show the child had lived after birth, the supposition of suffocation during birth through the twisting of the umbilical cord, cannot of course be entertained. If any cause operating after birth had produced the effusion in the chest, it is impossible that the child should have been born dead.

4. *The death of the child was most probably due to strangulation,* through the cloth found on its neck, or simultaneously through the hinderance to respiration, and the injury to its head. This opinion is borne out by the apoplectic state of the vessels of the head, the large extravasation in the chest and head, the wound in the left foot, which could not have been produced in the womb or during birth; and which on account of the ecchymosis accompanying it, must have occurred during life.

[REMARKS. This case offers some important reflections in relation to the proofs of infanticide. We fully agree with the reporter in the first two conclusions; viz. the immaturity of the child, and the fact of its not having breathed or breathed but imperfectly: but we do not see that the *medical* data which he had to guide him, justified so exclusive an opinion of this child having necessarily lived after birth. Lividity and ecchymosis about the neck and scalp offer no evidence of live birth; for experience shows that these may be equally produced during birth, and the child not come into the world alive. The fissure in the parietal bone is assumed to have been indicative of violence, merely because the bones were elastic and yielding; but, from the description of it, we are inclined to believe that it was probably a fracture from the efforts of the uterus during birth. Murderous violence to the head after birth is rarely confined to the production of a *slight fracture* in one parietal bone. At any rate, in the absence of direct evidence as to the origin of so slight an injury, the presumption is as strong for natural as for violent causes. The reporter seems to imagine that extravasation of blood can only take place in the *living* child; but it is well known that precisely similar appearances may result from violence to the body of one which is recently dead. The presence of extravasation or lividity cannot therefore be taken in an abstract view, as any evidence of live birth. The injury on the foot bore more clearly the marks of design; and this, together with the violence about the head seems to show, that the whole of the body of the child was in the world when it was produced; but whether the child was actually living or recently dead at the time, it does not establish. Perhaps the best evidence of this child having been living and wilfully destroyed, we have in the circumstances, especially in the fact that a cloth was tied tightly round the neck; for why should this be done to a child already dead? At the same time, this murderous violence might have been used towards it *during the act of birth.* The non-inflation of the lungs is of course no objection to the child having come into the world alive, and lived long enough to be the subject of murder.]

*Henke's Zeitschrift.* 1838.

---

*Fatal Wound of the Head. Death through alleged medical neglect from the non-performance of a necessary operation.* By Dr. SPEYER, of Bamberg.

ON the 19th March, 1837, a drunken man during a quarrel at an inn received a smart blow on the forehead from a stick. The shock was so severe that he was knocked down, remained insensible for half an hour, and vomited several times. A surgeon was called, who found by a cursory examination that the frontal bone above the left eye had been driven in, and having applied a bandage he directed that the man should not be moved from the house. In spite of this direction the man's friends in the course of the evening placed him in a waggon and drove him some miles over a rough and stony road. A nearer examination of the wound was made the following day. Its situation was on the left side of the frontal bone near its middle; it was about three quarters of an inch long, and the edges were ragged and depressed. The bone was indented, and at the lower part of the wound was evidently fissured, the fissure extending towards the nose. Vomiting had then returned, and there was so little consciousness that the necessary legal questions could not be put to him. The pulse was quick and other symptoms of febrile irritation had set in. Another surgeon, who was called in about this time, after having examined the wound, proposed trephining; but the operation was not performed, and on the 23d symptoms of inflammation of the brain appeared. Under these circumstances the operation was altogether declined, it being then considered too late to resort to it. The man continued to become worse, and on the 28th (the ninth day after the injury) he expired in violent convulsions. During the time that he lived the treatment had been chiefly of an antiphlogistic nature.

An inspection of the body was ordered, and attention was first directed to the situation of the wound. The bone was found indented and fissured: on raising it the internal table corresponding to the seat of injury was much fractured, the sharp spiculæ projecting into the dura mater which had been perforated to the extent of two lines in length and one in breadth. The brain had become here compressed and its substance inflamed. The whole of the vessels around were much congested. A small quantity of matter was found between the cortical substance and pia mater; and the left hemisphere was to some extent softened and pulpy. The other cavities of the body were successively examined, but, with the exception of a congested state of the upper and posterior parts of the lungs (probably cadaveric), nothing abnormal was found.

The medical opinion expressed was that death was a consequence of the wound, this having given rise to the fatal inflammation, suppuration, and softening of the brain. The inspectors then proceed to enquire whether these serious consequences operated by producing apoplexy or asphyxia, and at last settle that it must have been a case of *asphyxial apoplexy:* i. e. death must have proceeded partly from one and partly from the other condition. In relation to the treatment pursued, they suggested that as the fractured portion of the skull was the main source of irritation and death, this ought to have been removed by the trephine. They do not undertake to say that this operation would have necessarily been successful; but they thought that the deceased's recovery was possible only by its having been timely performed.

It next became necessary to consider how far the treatment pursued and the neglect to perform the operation had led to a fatal result. The removal of the deceased many miles soon after the accident must have rendered his condition more serious, since it favoured the access of inflammation. The proper time for the operation was, in their opinion, on the first or at the furthest on the second day; because by delay the result of the operation always became more uncertain. The legal questions were therefore answered in the following way:

1. The deceased died a violent death.

2. Death was not a direct but an indirect consequence of the wound. The wound could not be pronounced absolutely mortal, since a timely operation might have saved life; but the brain having sustained concussion from the violence, it was under all circumstances an injury dangerous to life. The law authorities not being satisfied required an answer from the Medical College of B., to the following question: "How far and in what degree of probability or of certainty the performance of the operation, or the application of proper treatment to the case, would have saved the life of the deceased?" They returned answers to the following effect:

1. The deceased died a violent death from the wound which he received on the head. The facts of the case sufficiently establish this.

2. The wounds were necessarily fatal. The deceased suffered from concussion as well as from compression and its consequences; all injuries of a most serious description. With the exception of drunkenness at the time of the accident, and his rough removal soon afterwards, there were no circumstances in the deceased's case likely to aggravate his condition. At the same time, the injury to the head was such that it might have readily destroyed life even had not these conditions existed.

3. The injuries were not of such a nature as necessarily in all cases to lead to death. Although he was seriously injured, it is possible that the deceased might have recovered under proper treatment. The great source of danger was the depressed bone leading to inflammation. Such a danger could only have been avoided by the timely use of the trephine. The operation was here imperatively called for; and it is in the highest degree probable that, had it been performed at a proper time, the life of the deceased might have been saved. Whatever difference of opinion may exist among surgeons as to the indications for this operation and the period for undertaking it, there could exist no doubt that it was eminently required in the case of the deceased. The opponents of the operation allow that it is necessary when, under fractures of the cranium, serious symptoms begin to appear. Had it been performed, judging from the healthy state of the deceased's body and his previous good health, it would probably have terminated favorably. Subsequent treatment might have removed the effects of the concussion.

4. The violence operated indirectly in causing death, namely, by exciting inflammation.

[REMARKS. This case shows the importance of carefully attending to the treatment of a person who has been the subject of criminal violence. We cannot hesitate to agree to the opinion expressed by the members of the college, that in not performing the operation at a seasonable period, all chance of the man's recovery was taken away. The deceased might have died even had it been performed, but the law very properly will not be governed by mere possibilities. Here was clear evidence to show that the best means for recovery had not been used, in accordance with good professional practice and experience; and therefore the prisoner could not be held responsible for the deceased's death, without in some sort punishing him for want of skill in the surgeon. In the "*superarbitrium*" of the college, the second and third positions appear contradictory and inconsistent with each other; but it is probable that they intend to draw a distinction between such wounds being necessarily fatal when improperly treated, as in the deceased's case, and not necessarily fatal when proper means have been timely resorted to. It is singular that our continental brethren are rarely contented with finding out a clear cause of death, but that they always endeavour to rise through this to a higher stage of causation. In the case before us, to all practical men the discovery of the mischief in the brain would have been considered as sufficient to account for death; but the reporters go still further,—they gravely enquire whether this mischief brought on apoplexy or asphyxia, and resolve that both conditions existed, because the vessels of the brain and lungs were somewhat congested. This kind of speculation, the offspring of transcendental physiology, is as profitless as it is absurd: we wish to see it wholly laid aside in sober medico-legal reports.]

*Henke's Zeitschrift.* 1838.

---

*Case of Suicidal Strangulation.* By Dr. CARGANICO, of Darkehmen.

A PEASANT was found lying dead close to a well-frequented road and in the neighbourhood of dwelling-houses. The following facts came to light from the judicial and medical examination.

The body was lying stretched out at full length on the abdomen, the arms placed at the sides, and the hands half-closed. There was no mark of violence on the person or dress, or appearance of struggling or trampling on the grass around. The deceased appeared to be a robust man of about fifty; the countenance was pale, with the exception of slight cadaverous ecchymosis about the root of the nose. The eyes and mouth were naturally closed, the lips pale, the tongue in its natural position not swollen, the ears not discoloured, the features having a very placid expression. The hands which were half-closed, as is usual in death, presented no traces of violence. Particular attention was now paid to the neck: the cravat worn by the deceased was found carried twice round the neck in the customary way, and tied by a knot in front. On the left side a small stick about four inches and a half long had been thrust between the two folds of the cravat, then twisted by a half-turn, and so brought round that the lower end rested firmly against the angle of the jaw, which prevented it from returning. This had so compressed the neck by tightening the cravat, that it was impossible to introduce a finger under it. Near the body a branch of a tree was found, from which the stick had evidently been cut.

The neck beneath the cravat was scarcely depressed, and the skin was free from all sugillation or desiccation. The larynx, trachea, and os hyoides were normal. The body was free from all traces of violence, and there was not even the least sign of cadaverous lividity. Being satisfied from these facts that the deceased had destroyed himself by strangulation, the reporter did not make an internal inspection, but proceeded to draw up a medical opinion. He observes that a case of such interest was deserving of closer examination, in which we fully agree with him; but, at the same time, he assigns no satisfactory reason for having omitted to institute it.

1. The *cause of death* was apoplexy, not asphyxia, i. e. the ligature did not act by interrupting respiration, but by compressing the cervical vessels and preventing the free circulation of blood through the brain. The proofs of this were: paleness of the countenance and lips, and absence of lividity in the body. The want of a well-defined and ecchymosed depression in the course of the ligature is an additional proof that asphyxia was not the cause; while in apoplexy from strangulation this ecchymosed depression is often wanting. The absence of a mark he satisfactorily accounts for, by the fact that the compressing material was soft and wide, and that it was not very tightly applied, although still sufficiently so to affect the cerebral circulation.

2. *Was the strangulation suicidal?* This was rendered in the highest degree probable, if not certain. Suicidal strangulation is not very common, but in this case the means employed for accomplishing it as well as the whole of the circumstances were only intelligible upon such a presumption. Strong corroborative proofs existed in the absence of violence to the person and dress, the position of the body, and placidity of the countenance. A healthy strong man like the deceased could not have been easily destroyed by others in the manner described, without indubitable evidence of the fact being left on his person. Again, the ligature to the neck had not been violently applied as it would have been by a murderer, but the compression of this part had been gradual and comparatively slight. Murder by strangulation would have been indicated by extensive injury to the skin, and probably to the deep-seated organs of the neck, at least this is what is commonly observed: but none of these signs existed in the case of the deceased.

[REMARKS. Two facts are we think pretty clear in this case, and they are fortunately all that the law requires to be established: 1, that the deceased died from strangulation; 2, that he strangled himself. Whether the ligature operated by impeding the cerebral circulation by preventing respiration, or in both ways, is a point of no importance in a judicial light, and highly absurd to enquire into as a medical fact, where the *exterior of the body* only has been seen. Casper's observations already reported in this Journal, establish that the production or non-production of an ecchymosed mark by a ligature in strangulation, can furnish no evidence as to whether death took place by apoplexy or asphyxia; and we think it would have been somewhat surprising to have met with ecchymosis under the circumstances of the present case. The reporter first assumes that the deceased died from apoplexy, next that the suicides who hang themselves die more frequently from this cause, and then he comes to the conclusion that the deceased's having died of apoplexy is a proof of his having committed suicide! There is fortunately good evidence of suicide in the facts themselves, thus rendering it unnecessary to resort to such weak hypothetical reasoning as this.]

*Henke's Zeitschrift.* 1838.

